# Levels of Physical Activity in the Adult Population of La Guajira, Colombia: A Focus on Ethnicity

**DOI:** 10.3389/fpubh.2020.610679

**Published:** 2021-02-04

**Authors:** Yaina Panciera-di-Zoppola, Juan Niño-Restrepo, José Melo-Freile, Rocío Ortiz-Moncada

**Affiliations:** ^1^Department of Community Nursing, Preventive Medicine and Public Health, History of Science, University of Alicante, Alicante, Spain; ^2^Faculty of Education, Bachelor's Degree in Physical Education, Recreation and Sports, University of La Guajira, Riohacha, Colombia; ^3^Guajira Research Group, University of La Guajira, Riohacha, Colombia; ^4^Food and Nutrition Research Group (ALINUT), University of Alicante, Alicante, Spain; ^5^National Training Service (SENA), Caldas, Colombia; ^6^Faculty of Engineering, University of La Guajira, Riohacha, Colombia

**Keywords:** physical activity, sedentary behavior, indigenous people, ethnic groups, demography

## Abstract

The objective of this study is to analyze the physical activity levels among the ethnic groups in La Guajira, Colombia, according to the different ethnic groups and their sociodemographic factors. With regards to ethnic groups, two groups were studied, ethnic (Indigenous and Afro-Colombian) and non-ethnic (White or Mestizo). In order to obtain the relevant data a non-probability sample of 251 people of ≥18 years of age were asked to complete the short version of the International Physical Activity Questionnaire (IPAQ). In this analysis, physical activity (METs.min^−1^) levels were considered as the dependent variable, while the independent variables were linked to the sociodemographic factors: sex, age, social class, civil status, educational level, and municipality of residence. The individuals were then categorized based on their physical activity levels and their compliance with the World Health Organization's (WHO) physical activity recommendations. Next, using the sociodemographic variables, regression models were made to determine the likelihood of the participants meeting these physical activity targets; these models found that 78.1% of the studied participants met the targets. According to the results of the bivariate analysis, participants of ≥47 years of age, and those with only a primary education presented a lower probability of complying with the physical activity recommendations, while those who lived in large municipalities (Riohacha) displayed a larger probability of compliance [OR = 2.16; 95% CI: (1.18–3.96)]. Furthermore, the multivariate analysis showed that Indigenous and Afro-Colombian people in a low social class are more likely to comply with the physical activity recommendations [ORad = 4.88; 95% CI: (1.31–18.1)], while residing in a smaller municipality (Manaure) is associated with a lower probability of compliance [ORad = 0.39; 95% CI: (0.16–0.91)]. In addition, the educated participants were shown to be active and to comply with the physical activity recommendations is to a high degree, even when compared with national and global results. In the ethnic group however, low social class stood out as the sociodemographic factor most associated with high physical activity, while Afro-Colombians were shown to be more active than the Indigenous people. The results of this study offer evidence that can either form a base for future research.

## Introduction

Physical activity is defined as the point between the highest expenditure of energy in any action that an individual performs, and the expenditure of energy when they are in a state of rest (1). Public health promotion strategies always recommend physical activity for the improvement of health, as well as for the primary and secondary prevention of chronic non-communicable diseases (2). However, despite this, one-third of the world's population is categorized as inactive or sedentary. This has lead to one of the largest public health crises in the world with a deterioration of lifestyle, and an increase in the prevalence of obesity, cardiovascular disease, and diabetes (3). Therefore, many countries have implemented regulations and legislation that promotes physical activity and encourage a healthy lifestyle (4).

While the health benefits of physical activity are recognized however, it is the frequency and intensity with which it is practiced that determines the amount needed to promote good health (4). In order to estimate the varying levels of physical activity among different populations, the WHO proposed the use of the metabolic equivalent of task (MET) as a unit for the measurement of the intensity of physical activity. The MET unit is defined as the energetic cost involved in an act, for example sitting and being calm is equivalent to 1 Kcal/kg/h, while a moderately active person would use three to six times more energy (3–6 METs), and a person engaged in vigorous activity uses more than six times that amount (>6 METs) (5). This unit of measurement has allowed for the homogenization of studies on the measurement of physical activity in across different populations (6–10).

Based on the findings from various international studies on physical activity among ethnic minorities -related to Indigenous communities, those of African descent, Romani groups, immigrants, race and religious groups–, minority ethnic groups are considered less active than the majority of the population, while the factors that influence their behavior are also different. In the North American continent, different dynamics have been observed among different ethnicities, for example, physical activity works as more of a protective factor, in terms of its protective qualities against cardiovascular diseases, among Indigenous people with higher activity levels than among the White population (11, 12). Furthermore, other indigenous groups, such as African-Americans, were shown to be more sedentary than others (13). A systematic review indicated that physical activity among ethnic minority groups in Europe meanwhile, is influenced by a wide variety of factors, including sex, religion, cultural requirements, and knowledge, and that these recurrent sociodemographic factors are part of the social/cultural environment as well as being part of various psychosocial factors (14). In countries like Australia, the dynamics of the Indigenous population's physical activity are explained from a socioecological perspective, where the surrounding environmental factors cannot be separated from social relationships, occupation, nutrition, or municipality of residence (15). In Canada, the sociocultural factors that determine the physical activity among Indigenous youth (16) are considered to be the cultural physical activities related to traditional games and cultural expressions that allow for participation within the community (17). On the Polynesian islands, sociocultural factors are explained from a collective perspective in which social organization favors collective activities over individual activities; a choice that is also reflected in the behaviors around physical activity and nutrition (18). In general, from an ethnicity perspective, studies point to differences in physical activity that could be explained by social determinants of health, such as social behavior (community surroundings, health care), or by influential sectors in the community (education, public health, forms of transport) (19). In the same way, although physical activity constitutes a protective on factor against cardiovascular disease, this does not carry over to other non-chronic diseases, such as type 2 diabetes; something which could be due to genetic differences in the physiology of some ethnic groups, such as in South Asians (12).

In Latin America and the Caribbean, both Indigenous people and those with African descent are categorized as ethnic minorities (20). Ethnicity is defined as a human group that shares and recognizes its own specific cultural characteristics, which determine its identity (ethos) and thus differentiates it from other groups (21). The scarce number of studies that exist on physical activity among these Indigenous communities in Latin America, show that their physical activity levels are relatively low or similar to the non-indigenous people. These results could possibly be explained by the transition that Indigenous populations are experiencing with their integration into the market, as these also interact with other historical, cultural, social, economic, environmental, and demographic factors; as has been the case in Chile and Ecuador. The study carried out in Chile identified small differences in the intensity of different indigenous groups' (Pehuenche and Atacameña) physical activity levels, while in Ecuador (the indigenous Shuar population), the study showed relatively low levels of physical activity. In both of these cases however, the physical activity levels could be explained by the assimilation of these communities into salaried jobs, an increase in subsistence activities and the market economy (22, 23).

Colombia particularly, is characterized by being a multi-ethnic and multilingual country that shows displays an important recognition of its minority groups (21). The findings by the National Health and Nutrition Survey (24), showed that 21.5% of the general population carry out physical activities in their leisure time. With regards to the ethnic group, the Indigenous population showed a significantly higher compliance with the physical activity recommendations (63%) than the Afro-Colombians and non-ethnic group (24). However, in Colombia, despite the development of regional plans and programs promoted by the government to stimulate physical activity as a means of social welfare, there is still no disaggregated information on the physical activity levels among regional ethnic groups. This is the case even in La Guajira, where a total concentration of 97.5% of Colombia's indigenous Wayuu population reside (25). The objective of this study is to analyze the physical activity levels among the ethnic groups in La Guajira, Colombia, according to the different ethnic groups and their sociodemographic factors.

## Materials and Methods

### Type of Study

A cross-sectional population study was conducted on the physical activity levels among the adult Colombian of La Guajira.

### Population and Sample

According to Colombia's Agustín Codazzi Geographic Institute, La Guajira is one of the political-administrative division's 32 departments (26). The department of La Guajira has a total population of 825,364 of which 53% are considered adults, and it is divided into 15 municipalities According to census records from 2018, the region's population is predominantly composed of ethnic groups, with 44.9% being indigenous Wayuu and 7.3% being Afro-Colombian, Palenquera and Raizal. Furthermore, the census shows that the highest concentration of Indigenous and Afro-Colombian populations are in the municipalities of Manaure, Maicao, Uribia, Riohacha, Barrancas, and Dibulla (21, 25).

For this study, a non-probability sample of 251 individuals resided across three municipalities, that contained approximately 21% of the adults in La Guajira, was taken (25). These municipalities were Manaure in Upper Guajira: (*n* = 74), Riohacha in Middle Guajira: (*n* = 151), and Barrancas in Lower Guajira: (*n* = 26). The criteria for the selection of municipalities were based on accessibility to the municipality, as these are not conflict zones or areas of public disorder.

Territorially speaking, these municipalities are characterized by their contrasting size, number of inhabitants, and total Indigenous population. Riohacha has 3,104 Km^2^ of land and has a concentration of 27.4% of the whole of La Guajira's population, 20% of which are Indigenous people. The next in size is Manaure, with 1,606 Km^2^, and a concentration of 11.1% of La Guajira's population, of which 62% are Indigenous people. Finally, Barrancas is the smallest municipality with only 670 Km^2^ to its name, and a concentration of 3.6% of La Guajira's total population, of which 26% are Indigenous people (27). For the benefit of the reader and to facilitate understanding, the municipalities will be referred to according to their territorial characteristics: Riohacha (large municipality), Manaure (medium municipality), and Barrancas (small municipality).

Inclusion criteria: individuals of ≥18 years of age, who understand Spanish and reside in urban areas (due to conflicts and areas of public disorder in rural areas). Pregnant women were excluded from this study.

### Study Variables

The response variable refers to the level of physical activity, as per the recommendations established by the WHO (5): light-level (≤600 METs.min^−1^ per week), moderate-level (601–3,000 METs.min^−1^ per week) and, vigorous-level (>3,000 METs.min^−1^ per week). The dependent variable was obtained from the answers from the participants' of the short version of the IPAQ (28). The responses obtained were transformed into METs (29) by means of the estimated numerical values standardized by Crespo-Salgado et al. (METs × minutes × days of the week) (30). Then, using this information, the participants were categorized as inactive or mildly physically active, moderately physically active and vigorously physically active.

The independent variables were related to personal and sociodemographic characteristics, and ethnic group. The personal and sociodemographic variables were: Sex (woman, man); age (age groups 18–26; 27–36; 37–46, 47–56; 57–66; ≥67 years); civil status (single, married, common-law marriage or registered partnership, separated); social class [categorized according to the strata of residential properties that receive public services, with 6 being the wealthiest stratum and 1 being the poorest (31)]; educational level (doctorate, master's degree, bachelor's degree, technical qualifications, high school, middle school, and primary school); and municipality of residence (territorial size), referring to the municipality where the respondent resided at the time of the interview, specifically Riohacha (large municipality), Manaure (medium municipality), and Barrancas (small municipality). The group considered as the ethnic group, was formed directly from the variables determined by the Colombian government's National Administrative Department of Statistics (DANE) and in this way respected their classification (asking: Which ethnic group do you self-identify as? Afro-Colombian, Indigenous, White, Mestizo). The White and Mestizo population is classed as non-ethnic (25).

### Instruments Used in the Collection of Information

A two-part questionnaire was put together with a total of 14 questions. The first part consisted of seven Likert scale questions formed using questions drawn from national and regional questionnaires on socioeconomic aspects (31, 32). The second part consisted of the short version of the IPAQ (validated) which presents seven questions related to the practice of daily physical exercise (28, 33).

The questionnaire was distributed to the participants during an interview by three nutrition experts who specialize in physical activity and have local knowledge of the municipalities. This last requirement was essential to facilitate access to the interviewed individuals, and to ensure that the interviewers were in places without armed conflict. The group of field professionals selected the participants in each municipality through a consensus. The interviews were conducted between March and June 2018 and lasted approximately 20 min each.

### Analysis and Interpretation of Data

For the interpretation of data, the sociodemographic variables were re-categorized: 1. Age (individuals of 47–56, 57–66, 67–76, and ≥77 years of age were grouped into one category of ≥47 years of age); 2. civil status (single and separated individuals were grouped into one category); 3. social class [medium (strata 5 + 4 + 3 = ≥3), low (stratum 2), and very-low (stratum 1); and 4. educational level (individuals with higher educational levels were grouped into university + postgraduate].

To assess the factors associated with the level of physical activity, the recommendations established by the WHO were taken as the gold standard (5). For the interpretation of the results, the physical activity recommendations were grouped according to compliance “YES” or non-compliance “NO.” The people who fulfilled the recommendations were classified as those who were active, participating in >600 METs.min^−1^ per week, while the non-compliant group consisted of those who were inactive, participating in ≤ 600 METs.min^−1^ of physical activity per week.

In addition, in order to evaluate the population participants' physical activity levels among both those who are considered socially vulnerable minorities (e.g., Indigenous people) and those considered as the majority population (e.g., Whites) (34), the studied population was re-categorized into: ethnic groups (Indigenous people + Afro-Colombians) and non-ethnic groups (White + Mestizo). In order to correctly interpret the results, both the completion of the physical activity recommendations (>600 METs.min^−1^ per week) and the sociodemographic characteristics were taken into consideration for both groups.

The descriptive analysis used the sociodemographic variables to measure the frequency with which physical activity was undertaken (absolute and relative) in each category, across the total sample. The chi-square test with a statistical significance level of *p* < 0.05 was performed alongside the Odds Ratio (OR) method, in order to observe the differences between the studied groups according to their compliance with the physical activity recommendations. Binary logistic regression techniques (ORcrude) and 95% confidence intervals (CI) were calculated, oriented toward the probability of compliance with the physical activity recommendation, and then this was stratified according to the ethnic and non-ethnic groups for each sociodemographic variable studied. In addition, an adjusted multivariate analysis (ORa) for potential confounding factors was performed on: Sex, age, marital status, socioeconomic level, educational level, and municipality of residence. The analyses were performed with the statistical package IBM-SPSS Statistics for Windows, version 26.0.

### Ethical Aspects

The informed consent and ethical permission are both included in the questionnaire, as per the Colombian regulations and the Helsinki declaration that regulate any research carried out in the health field (35, 36).

## Results

Table 1 shows the distribution of the sociodemographic characteristics among the studied population. The results show that 57% of the participants are women, 79.3% are under the age of 46, 33.1% identify as single, 23.9% have a primary education, and 60.2% live in the capital of the department (Riohacha). With regards to social class, 90% are of a low social class, in social strata 1 and 2 (19.1 and 70.5%, respectively), and there is an absence of people in the highest social strata (5 and 6). Furthermore, 69.7% belong in the ethnic group, specifically Afro-Colombians (39%) and Indigenous people (30.7%).

**Table 1:** Distribution of the sociodemographic characteristics of the study population of La-Guajira, Colombia.

**Variables**	***n***	**%**
**SEX**		
Woman	143	57
Man	108	43
**AGE (YEARS)**		
18–26	57	22.7
27–36	79	31.5
37–46	63	25.1
47–56	31	12.3
57–66	17	6.80
≥67	4	1.60
**MARITAL STATUS**		
Single	83	33.1
Married	55	21.9
Separated	19	7.50
Common-law Marriage	94	37.5
**SOCIAL CLASS (STRATA)**		
6	0	0
5	1	0.40
4	3	1.20
3	22	8.80
2	177	70.5
1	48	19.1
**LEVEL OF EDUCATION**		
Master - Doctorate Degree	23	9.20
Bachelor's Degree	20	7.90
Technical Qualifications	66	26.3
Middle - High School	82	32.7
Primary School	57	23.9
**MUNICIPALITY OF RESIDENCE (TERRITORIAL SIZE)**		
Riohacha (large)	151	60.2
Manaure (medium)	74	29.5
Barrancas (small)	26	10.4
**ETHNIC GROUP**		
Indigenous People	77	30.7
Afro-Colombians	98	39.0
Non-ethnic Group	76	30.3
**Total**	**251**	**100**

Table 2 shows the physical activity levels according to the studied population's sociodemographic factors. The results indicate that 47% have a moderate physical activity level and 31.1% have a vigorous level, with the best levels found in men and Afro-Colombians (when compared to others within the ethnic group). The most prevalent moderate physical activity level is found in 54.4% of those between the ages of 27–38, 63% among those who are married, 56.1% of those with a secondary education, and 71.5% of those in social stratum 1.

**Table 2:** Levels of physical activity according to the sociodemographic factors of the population of La-Guajira, Colombia.

**Variables**	**Level of physical activity**					
	**Light**^**a**^		**Moderate**^**b**^		**Vigorous**^**c**^	
	**METs.min^**−1.**^week**	***n* (%)**	**METs.min^**−1**^.week**	***n* (%)**	**METs.min^**−1**^.week**	***n* (%)**
**SEX**						
Woman	350.9 ± 143.5	38 (26.6)	1,604.8 ± 679.4	66 (46.1)	4,462.3 ± 1,472.9	39 (27.3)
Men	365.4 ± 131.9	17 (15.7)	1,725.8 ± 614.6	52 (48.1)	4,699.1 ± 1,792.8	39 (36.2)
**AGE (YEARS)**						
18–26	422.7 ± 129.1	10 (17.5)	1,555.6 ± 724.6	25 (43.9)	4,408.2 ± 1,262.9	22 (38.6)
27–36	308 ± 160.2	15 (19.0)	1,538.1 ± 672.4	43 (54.4)	4,590.1 ± 1,800	21 (26.6)
37–46	383.8 ± 119.3	13 (20.6)	1,888.2 ± 580.81	30 (47.6)	4,707.5 ± 1,735.6	20 (31.7)
47–56	362.7 ± 126.68	14 (45.1)	1,702.9 ± 567.7	13 (41.9)	4,176.5 ± 1,046.2	4 (12.9)
57–66	225 ± 46.8	3 (16.7)	1,692.3 ± 569.6	7 (38.9)	5,306.1 ± 2,320.6	8 (44.4)
≥67	0	0	0	0	3,539.3 ± 169.2	3 (100)
**MARITAL STATUS**						
Single	374.6 ± 153.25	19 (24.7)	1,704.5 ± 721.5	42 (54.5)	4,065.3 ± 1,296.8	16 (20.8)
Married	373.4 ± 123.3	10 (21.8)	1,537.6 ± 599.4	29 (63)	4,531.1 ± 1,844.8	7 (15.2)
Separated	346.5 ± 188.8	2 (5.60)	1,884.7 ± 595.3	12 (33.3)	4,202.8 ± 849.6	22 (61.1)
Common-law Marriage	333.522 ± 133.8	24 (26.1)	1,652.2 ± 621.2	35 (38)	5,093.2 ± 2,006.1	33 (35.9)
**SOCIAL CLASS (STRATA)**						
5	462	1 (100)	0	0	0	0
4	288 ± 12.7	2 (66.7)	2,148	1 (33.3)	0	0
3	293.1 ± 134	7 (25.0)	1,839.5 ± 672.1	10 (35.7)	3,739 ± 820.1	11 (39.3)
2	363.7 ± 141.4	38 (21.5)	1,626.9 ± 641.2	77 (43.5)	4,804.7 ± 1725.5	62 (35.0)
1	383 ± 148	7 (16.6)	1,661.4 ± 688.3	30 (71.5)	3,656 ± 733.1	5 (11.9)
**LEVEL OF EDUCATION**						
Master - Doctorate Degree	413 ± 86.4	4 (17.4)	2,206.8 ± 748.5	11 (47.8)	4,264.9 ± 1,015.3	8 (34.8)
Bachelor's Degree	299 ± 189.4	6 (30.0)	1,727.3 ± 761.6	7 (35.0)	4,457.1 ± 1,445.6	7 (35.0)
Technical Qualifications	356.1 ± 147.9	9 (13.7)	1,526.1 ± 688.8	35 (53.0)	4,169.8 ± 1,418	22 (33.3)
Middle - High School	355.4 ± 136.8	13 (15.9)	1,597 ± 540.8	46 (56.1)	4,917.7 ± 1,864.1	23 (28.0)
Primary School	361.7 ± 134.8	23 (38.3)	1,706 ± 630.3	19 (31.6)	4,840.9 ± 1,859.6	18 (30.0)
**MUNICIPALITY OF RESIDENCE (TERRITORIAL SIZE)**						
Riohacha (large)	336.7 ± 125.8	25 (16.6)	1,777.3 ± 621.6	74 (49.0)	4,545.9 ± 1,606.6	52 (34.4)
Manaure (medium)	412.1 ± 136.9	21 (28.4)	1,552.2 ± 667.3	30 (40.5)	4,722.3 ± 1,796.7	23 (31.1)
Barrancas (small)	279.8 ± 137.9	9 (34.7)	1,255.1 ± 614.9	14 (53.8)	4,099.3 ± 946.8	3 (11.5)
**ETHNIC Group**						
Indigenous	353 ± 133.5	24 (31.1)	1,688.7 ± 648	32 (41.6)	4,824.5 ± 1,864.8	21 (27.3)
Afro-Colombians	337.7 ± 157.4	19 (19.4)	1,696.5 ± 638.8	48 (49.0)	4,596.3 ± 1,590.8	31 (31.6)
Non-ethnic Group	391.8 ± 119.3	12 (15.8)	1,584 ± 681.5	38 (50.0)	4,365.3 ± 1,519.7	26 (34.2)
Total	356.1	55 (21.9)	1,658.1	118 (47)	4,580.7	78 (31.1)

a *Light level (≤600 METs.min^−1^ per week)*.

b *Moderate level (601–3,000 METs.min^−1^ per week)*.

c *Vigorous level (>3,000 METs.min^−1^ per week)*.

Table 3 shows the level of compliance with physical activity recommendations and its association with sociodemographic characteristics. It points to the fact that 78.1% of the studied population complies with the physical activity recommendations and that age (*X*^2^ = 4.45; *p* = 0.035), educational level (*X*^2^ = 12.42; *p* < 0.001), municipality of residence (*X*^2^ = 6.35; *p* = 0.012), and ethnicity all impact the levels of that compliance. Through this, it can be observed that the participants of ≥47 years of age [OR = 0.49; 95% CI: (0.25–0.96); *p* < 0.05] and those that have a primary level education [OR = 0.32; 95% CI: (0.17–0.62); *p* < 0.05], have a lower probability of complying with the physical activity recommendations, that is to say, they are less active. Furthermore, the results show that those living in Riohacha (large municipality), as opposed to those living in Manaure (small municipality) and Barrancas (medium municipality), have higher activity levels and a higher probability of complying with the recommendations [OR = 2.16; 95% CI: (1.18–3.96); *p* < 0.05]. Finally, with regards to ethnicity, it is observed that the studied Indigenous community, as opposed to the Afro-Colombians and non-ethnic groups, are less active and have a lower probability of complying with the recommendations [OR = 0.48; 95% CI: (0.26–0.89); *p* < 0.05].

**Table 3:** Compliance with the physical activity recommendations and its association with the sociodemographic characteristics of the population of La-Guajira, Colombia.

**Variables**	**Compliance With physical activity**		**OR (CI 95%)**
	**Recommendations** ***n*** **(%)**		**Compliance (YES)**
	**NO (Inactive^**a**^)**	**YES (Active^**b**^)**	
**SEX**			
Woman	38 (26.6)	105 (73.4)	0.53 (0.28–1)
Man	17 (15.8)	91 (84.2)	1.89 (1–3.59)
**AGE (YEARS)**			
18–26	10 (17.5)	47 (82.5)	1.41 (0.66–3.03)
27–36	15 (19.0)	64 (81.0)	1.29 (0.67–2.51)
37–46	13 (20.6)	50 (79.4)	1.11 (0.55–2.23)
≥47	17 (32.7)	35 (67.3)	0.49 (0.25–0.96)*
**MARITAL STATUS**			
Single + separated	21 (20.6)	81 (79.4)	1.14 (0.61–2.11)
Married	10 (18.2)	45 (81.8)	1.34 (0.63–2.87)
Common-law marriage	24 (25.5)	70 (74.5)	0.71 (0.39–1.32)
**SOCIAL CLASS (STRATA)**			
Medium-high (strata ≥ 3)	10 (38.5)	16 (61.5)	0.49 (0.19–1.20)
Low (strata 2)	38 (21.5)	139 (78.5)	0.91 (0.47–1.78)
Very low (strata 1)	7 (14.6)	41 (85.4)	1.87 (0.79–4.43)
**LEVEL OF EDUCATION**			
University + Postgraduate	10 (23.3)	33 (76.7)	0.91 (0.42–1.99)
Technical Qualifications	9 (13.6)	57(86.4)	2.09 (0.96–4.56)
Middle - High School	13 (15.9)	69 (84.1)	1.75 (0.88–3.49)
Primary School	23 (38.3)	37 (61.7)	0.32 (0.17–0.62)*
**MUNICIPALITY OF RESIDENCE (TERRITORIAL SIZE)**			
Riohacha (large)	25 (16.6)	126 (86.4)	2.16 (1.18–3.96)*
Manaure (medium)	21 (28.4)	53 (71.6)	0.6 (0.32–1.13)
Barrancas (small)	9 (34.6)	17 (65.4)	0.48 (0.2–1.16)
**ETHNIC GROUP**			
Indigenous People	24 (31.2)	53 (68.8)	0.48 (0.26–0.89)*
Afro-Colombians	19 (19.4)	79 (80.6)	1.28 (0.68–2.39)
Non-ethnic Group	12 (15.8)	64 (84.2)	1.73 (0.86–3.52)
Total	55 (21.9)	196 (78.1)	

a *Inactive: ≤ 600 METs.min^−1^ per week [according to the WHO (5)]*.

b *Active: > 600 METs.min^−1^ Per week [according to the WHO (5)]*.

* *Significant differences (p < 0.05)*.

*OR, Odds Ratio*.

Table 4 is disaggregated into ethnic (Indigenous and Afro-Colombian) and non-ethnic groups, and shows the association between compliance with the physical activity recommendations and the sociodemographic variables. The results indicate that compliance with the physical activity recommendations is associated to belonging to a low social class, particularly belonging to stratum 2 [ORa = 4.88; 95% CI: (1.31–18.1); *p* < 0.05]. The lower probability of compliance with the recommendations on the other hand, is associated with residing in Manaure [ORa = 0.39; 95% CI: (0.16–0.91); *p* < 0.05]. With regards to the non-ethnic group, a lower probability for compliance with the recommendations is shown by those who reside in Manaure [ORa = 0.05; 95% CI: (0–0.91); *p* < 0.05] and Barrancas [ORa = 0.005; 95% CI: (0–0.16); *p* < 0.05], when compared to those residing in Riohacha.

**Table 4:** Association between compliance with the recommendations of physical activity among the ethnic population of La Guajira and its sociodemographic characteristics, Colombia.

**Variables**	**Compliance With recommendations**			
	**Ethnic group**^**a**^		**Non-ethnic group**^**b**^	
	**OR (c)^**c**^**	**OR (a)^**d**^**	**OR (c)^**c**^**	**OR (a)^**d**^**
	**(95% CI)**	**(95% CI)**	**(95% CI)**	**(95% CI)**
**SEX**				
Woman	1 Ref.	1 Ref.	1 Ref.	1 Ref.
Man	1.54 (0.76–3.14)	1.13 (0.51–2.50)	1.55 (0.41–5.82)	3.14 (0.38–26.2)
**AGE (YEARS)**				
18–26	1 Ref.	1 Ref.	1 Ref.	1 Ref.
27–36	0.82 (0.29–2.29)	1.10 (0.34–3.60)	1.67 (0.24–11.13)	6.78 (0.2–251.6)
37–46	0.75 (0.26–2.17)	1.19 (0.34–4.20)	1.33 (0.2–9.02)	0.33 (0–14.3)
≥47	0.50 (0.17– 1.47)	1.10 (0.31–3.90)	0.33 (0.07–1.7)	0.03 (0–1.20)
**MARITAL STATUS**				
Single + separated	1 Ref.	1 Ref.	1 Ref.	1 Ref.
Married	1.29 (0.45–3.70)	1.67 (0.5–5.50)	1.02 (0.26–4.02)	32.9 (1.04–1040.7)
Common-law marriage	0.63 (0.3–1.35)	0.76 (0.32–1.81)	4.34 (0.49–38.3)	22.7 (0.54–959.7)
**SOCIAL CLASS (STRATA)**				
Medium-high (strata ≥ 3)	1 Ref.	1 Ref.	1 Ref.	1 Ref.
Low (strata 2)	2.38 (0.81–6.96)	4.88 (1.31–18.1)*	2.92 (0.57–14.9)	1.43 (0.09–21.5)
Very low (strata 1)	4.86 (1.13–20.84)*	4.61 (0.94–22.5)	2.28 (0.36–14.7)	1.42 (0.04–52.1)
**LEVEL OF EDUCATION**				
University + Postgraduate	1 Ref.	1 Ref.	1 Ref.	1 Ref.
Technical Qualifications	2.35 (0.71–7.81)	2.37 (0.63–8.91)	1.59 (0.23–11.2)	6.63 (0.28–158.7)
Middle - High School	2.01 (0.66–6.19)	1.87 (0.55–6.37)	1.25 (0.22–7.26)	17.30 (0.49–610.89)
Primary School	0.56 (0.19–1.64)	0.41 (0.11–1.57)	0.52 (0.1–2.85)	1.61 (0.07–34.6)
**MUNICIPALITY OF RESIDENCE (TERRITORIAL SIZE)**				
Riohacha (large)	1 Ref.	1 Ref.	1 Ref.	1 Ref.
Manaure (medium)	0.49 (0.22–1.03)	0.39 (0.16–0.91)*	0.39 (0.08–1.99)	0.05 (0–0.91)*
Barrancas (small)	0.69 (0.20–2.38)	0.35 (0.08–1.50)	0.09 (0.02–0.51)*	0.005 (0–0.16)*

a *Ethnic group: Indigenous people and Afro-Colombians*.

b *Non-ethnic group: White and Mestizo*.

c *ORc crude and 95% CI obtained through Binary Logistic Regression*.

d *ORa adjusted for all the independent variables included in the model (sex, age, marital status, social class, educational level, and municipality of residence)*.

*Ref., Reference value and condition*.

* *Indicates a significant difference from the reference condition (95% IC)*.

## Discussion

This cross-sectional study carried out on a sample of the adult Colombian population, of three different municipalities of a large, medium and small size in La Guajira, with and who's populations that included Indigenous people and Afro-Colombians (ethnic group) as well as White and Mestizos (non-ethnic group), shows that Indigenous people are less active than the Afro-Colombians. In addition, it shows that among the studied ethnic group belonging to a lower social class is associated with higher physical activity levels and that among both groups, residing in a municipality of a small size is associated with having a low physical activity level.

The physical activity levels among La Guajira's studied population could be considered excellent when compared to other regions of the world as well as when compared on a national level. The study shows that about half of the individuals (47%) engaged in moderate levels of physical activity and 31% engaged to a vigorous level. The percentage of individuals engaged in physical activity to a vigorous level in La Guajira was similar to that shown by the data on individuals at a global level (31.4%), but higher than those in North America (24.6%) and Europe (25.4%). There is also a higher prevalence of men than women showing a vigorous physical activity level; something that is common in this type of study (37). In this study, 78.1% of La Guajira's participants showed an active condition that was above the globally established 68.9% (37) and higher than other countries in the Americas such as the United States (10%) (38) and the Dominican Republic (59.2%), which are among the lowest. In contrast, the high levels of physical activity shown in the study population is similar to those reported by Chile 80.2% (8), Ecuador (77.4%), and Brazil (72.2%) (39). Furthermore, compared to the general Colombian population, the studied population in La Guajira showed higher physical activity levels than those reported in urban areas (51.1%), and even higher than those reported in Colombia's Atlantic region (45.9%) (24). According to the results of the 2015 National Nutrition Survey (24), this higher rate of engagement with physical activity is observed in both men and women, and is higher in La Guajira's population than the general Colombian population by 61.1 and 42.7%, respectively.

Correlating with the findings from this study, scientific literature shows that “age” is a global determining factor in the levels of engagement with physical activity, where physical activity decreases with age (37). A study in Chile showed increased physical activity among 86.6% of young people between the ages of 18–24, while a decreasing physical activity was observed in 62.8% of those over the age of 65 (8). In the same way, a study carried out in the United States showed that 63.9% of individuals between the ages of 20–29 were more compliant with physical activity recommendations while 46.9% of those over the age of 70 saw a decrease in their compliance with the recommendations (38). In addition, educational level was also shown, as in our research, to be another factor related to physical activity levels; the aforementioned studies showed that those who had undertaken basic primary education had a lower physical activity level when compared to those who had a moderate education [those with a secondary education showed and those with a higher education (8)].

In the present study, a higher engagement with physical activity was observed in the lower social class (stratum 2) than the higher social classes within the ethnic group. With this in mind, economic factors, and even cultural factors must be mentioned as having a possible role in influencing engagement with physical activity. For example, among the Polynesian islands' Indigenous population, it was observed that engagement with physical activity was not related to socioeconomic status but to more cultural factors, such as obesity and eating habits (18). Meanwhile, and in contrast to the present study, research carried out in Chile showed that there was a prevalence of lower physical activity levels among adults with a lower income (22.9%) when compared to those with a moderate income (18.4%) (8). While, another Latin American study, highlighted that it is the regions where the highest concentration of Indigenous people are, that have less access to information, sport, public services and recreation facilities (20). Thus, though economic and cultural factors play a role in people's engagement with physical activity, it seems that there is no pattern of association between physical activity and socioeconomic levels.

Global tendencies indicate that the prevalence of lower physical activity levels is higher in countries with a higher income (37–39). These results are similar to those found by this study, as when disaggregating the results of the ethnic and non-ethnic groups during the logistic regression analysis, it was observed that within the ethnic group, the lower social classes (stratum 2) complied more with the physical activity recommendations than those in a higher social class (stratum ≥ 3). A possible explanation to this phenomenon is the jobs that these individuals may have; manual jobs may have higher occupational physical activity demands when compared to non-manual jobs, with leisure time increasing in the same way it does for the groups with a higher income (40, 41).

There are some reports on physical activity among Indigenous populations that have been carried out under a demographic perspective that includes ethnicity. For example, a study in Canada compared the vigorous physical activity among Whites to the physical activity levels among different ethnicities (Aborigines, Blacks, Latin Americans, West Asians or Arabs, and South Asians). Through this, they observed that Aboriginal people were more likely to be physically active, while those from East or Southeast Asia were less likely to comply with the physical activity recommendations, thereby showing changes in physical activity according to ethnicity (11). These results are contrary to what was observed in this study, since although ethnicity showed an association to compliance with the physical activity recommendations, it was found that Indigenous people were less likely to be physically active when compared to the non-ethnic groups, as well as when compared to other ethnic groups. Furthermore, and again in contrast to this study, studies carried out in Australia found that the level of compliance with the recommendations among Aboriginals (63.15%) and non-Aboriginals (65.4%) did not present significant differences. In fact, they found that the studied characteristics associated with physical activity, such as healthy lifestyle habits, surrounding infrastructure (such as access to shops/services, public transportation, recreation facilities, etc.) and social relations, were generally very similar for both groups (42). Similarly to the Australian study, in Chile it was reported that there were no statistically significant differences in moderate-vigorous physical activity levels between the indigenous Mapuche people and those of European descent (43).

The current study with its focus on ethnicity, could be used as a reference for future research as it is one of our region's pioneering studies in this area. Among the limitations of the present study however, it must be noted that due to the use of a non-probabilistic sample, the interpretation of the results cannot cover the entirety of La Guajira's population. Furthermore, the presence of public disorder and conflicts in the region are also a barrier for the carrying out of randomized studies. However, in order to minimize possible errors, the municipalities with the greatest ease of access were randomly selected according to whether they belonged to Upper, Middle or Lower Guajira (44). Another limiting factor was the difference in sample size collected between the studied municipalities which was due to elements such as density of the population and the initiative with which individuals participated, which was greater in Riohacha. This discrepancy was minimized by the adjustment of any confusing factors through the employment of all the independent variables. Another of the study's limitations was the implicit subjectivity involved in the application for a survey (IPAQ self-report) to obtain the necessary quantitative data on physical activity levels in the area. This was controlled however by using “age,” due to its inverse relationship with physical activity—in that the higher the age, the lower the physical activity level—as a fundamental sociodemographic characteristic (37). In addition, it must be noted that the use of the standardized questionnaire continues to be the tool of greatest use for studies on populations (33).

The results also showed that the studied population in La Guajira complieds with the recommended physical activity, which could be a contributing factor to the prevention of pathologies associated to cardiovascular diseases and type 2 diabetes (2, 40, 43). Further to this, proposed population strategies, that include the creation of bio-health spaces with mechanical elements that facilitate physical activity, have been made with the aim to encourage an increase in physical activity (45). However, these strategies are not options for all the ethnic groups and are instead specifically designed for the Indigenous. In fact, Manaure's Territorial Health Plan encompasses the very problems that the municipality has of not having enough space to carry out activities aimed at those residing in urban areas, and neither does it have programs to save the customs and games of the indigenous Wayuu community, or give effective plans for the use of leisure time. Although the plan gives evidence that shows the necessities of these Indigenous people, it does not incorporate in its politics the use of physical activities as a cross-component for the promotion of health, in a way that would guarantee the strengthening of their culture and customs around the carrying out of traditional games, recreation and physical activities (46). With this in mind, the authors reflect on the implications of this study on public policies. Local institutions should continue to invest in stimulus programs that encourage increased physical activity with a specific approach for ethnic groups. In addition, studies on policy analysis in the region could be carried out to help understand the strategies associated with the promotion of physical activity and its impact on the population.

It is concluded that the studied Colombian population of La Guajira is active and their compliance with the physical activity recommendations is high, even when compared to national and global results. It is further noted that within the ethnic group, the Afro-Colombians are more active than the Indigenous community. In addition, among the sociodemographic factors, age, education, municipality of residence and ethnicity are the main sociodemographic factors that explain impact the physical activity levels among the studied population and the level to which they comply with the recommendations. The results show that being older, with a low level of education, and self-identifying as Indigenous, is associated with low physical activity and non-compliance with the physical activity recommendations, while residing in a large territorial municipality is associated with a higher level of physical activity. When disaggregating the results in the ethnic group, it was concluded that belonging to a low social class is associated with compliance with the physical activity recommendations among both Indigenous people and Afro-Colombians.

## Data Availability Statement

The raw data supporting the conclusions of this article will be made available by the authors, without undue reservation.

## Ethics Statement

Ethical review and approval was not required for the study on human participants in accordance with the local legislation and institutional requirements. The patients/participants provided their written informed consent to participate in this study.

## Author Contributions

YP and RO-M: conceptualization and methodology. JM-F: software. JM-F, YP, and RO-M: validation. YP and JN-R: formal analysis. YP: investigation, resources, writing – original draft preparation, project administration, and funding acquisition. YP and JM-F: data curation. RO-M: writing – review, editing, and supervision. All authors contributed to the article and approved the submitted version.

## Conflict of Interest

The authors declare that the research was conducted in the absence of any commercial or financial relationships that could be construed as a potential conflict of interest.
